# Biogeographic Patterns of Ectomycorrhizal Fungal Communities Associated With *Castanopsis sieboldii* Across the Japanese Archipelago

**DOI:** 10.3389/fmicb.2019.02656

**Published:** 2019-11-14

**Authors:** Shunsuke Matsuoka, Takaya Iwasaki, Yoriko Sugiyama, Eri Kawaguchi, Hideyuki Doi, Takashi Osono

**Affiliations:** ^1^Graduate School of Simulation Studies, University of Hyogo, Kobe, Japan; ^2^Department of Biological Sciences, Faculty of Science, Kanagawa University, Hiratsuka, Japan; ^3^Graduate School of Human and Environmental Studies, Kyoto University, Kyoto, Japan; ^4^Department of Life Science Frontiers, Center for iPS Cell Research and Application, Kyoto University, Kyoto, Japan; ^5^Department of Environmental Systems Science, Faculty of Science and Engineering, Doshisha University, Kyoto, Japan

**Keywords:** assembly process, biogeography, ectomycorrhiza, fungal community, host genotype, spatial structure

## Abstract

Biogeographic patterns in ectomycorrhizal (ECM) fungal communities and their drivers have been elucidated, including effects of host tree species and abiotic (climatic and edaphic) conditions. At these geographic scales, genotypic diversity and composition of single host tree species change with spatial and environmental gradients, reflecting their historical dispersal events. However, whether the host genotypes can be associated with the biogeographic patterns of ECM communities remains unclear. We investigated the biogeographic pattern of ECM fungal community associated with the single host species *Castanopsis sieboldii* (Fagaceae), whose genotypic diversity and composition across the Japanese archipelago has already been evaluated. ECM communities were investigated in 12 mature *Castanopsis*-dominated forests covering almost the entire distribution range of *C. sieboldii*, and we quantified the effect of host genotypes on the biogeographic pattern of ECM fungal communities. Richness and community composition of ECM fungi changed with latitude and longitude; these biogeographic changes of ECM community were significantly correlated with host genotypic variables. Quantitative analyses showed a higher relative explanatory power of climatic and spatial variables than that of host genotypic variables for the biogeographic patterns in the ECM community. Our results suggest historical events of host dispersal can affect the biogeographic patterns of the ECM fungal community, while their explanation power was lower than that for climatic filtering and/or fungal dispersal.

## Introduction

Ectomycorrhizal (ECM) fungi are symbionts of tree species, for example Fagaceae, Betulaceae, Dipterocarpaceae, and Pinaceae, which can be found in a wide range of forest ecosystems throughout the world (Brundrett, 2009). The ECM fungi are a major component of the forest floor and play an essential role in nutrient cycling via exchanging soil nutrients and therefore are critical for determining and maintaining forest ecosystem processes (Smith and Read, 2008). To infer the community responses to environmental changes, the relationships between spatial patterns of ECM fungal communities and factors responsible for those patterns have been investigated in previous studies (Lilleskov and Parrent, 2007). In these studies the spatial variations of ECM fungal community were related to key environmental factors including both biotic, such as species identity and phylogeny of the host (Ishida et al., 2007; Tedersoo et al., 2013), and abiotic factors, such as soil property (like soil pH) and climatic conditions (e.g., Bahram et al., 2012; Horton et al., 2013). At a more local scale, recent studies have shown that dispersal of ECM fungi is limited spatially, even at a few kilometers, and that this dispersal limitation can generate spatial structures in ECM fungal communities independent of environmental factors (Peay et al., 2012; Peay and Bruns, 2014). Thus far, the effects of these factors have been investigated at relatively small spatial scales, from forest to landscape scales (e.g., Tedersoo et al., 2011; Bahram et al., 2012; Matsuoka et al., 2016b).

Recently, in some pioneer studies the geographic distributions of ECM fungal communities at regional and/or continental scales were investigated (ranging from hundreds to thousands of kilometers). Species identity, foliar chemistry, and/or phylogeny of host trees are one of the most important factors generating geographic structures of ECM fungal communities (Põlme et al., 2013; Roy et al., 2013; van der Linde et al., 2018; Wu et al., 2018). Unlike free-living organisms, host-associated microbial communities like ECM fungi often exhibit biogeographic patterns that are related to the distribution of their hosts (Martiny et al., 2006), because of co-migration of microbes with their host species to new habitats (Kennedy et al., 2011; Séne et al., 2018). Therefore, these recent results imply that some part of ECM fungal communities respond to environmental change by following the movement of host trees. However, in studies at regional and/or continental scales, host tree compositions inevitably show the biogeographical structure reflecting both present environments and historical distribution and dispersal (e.g., Svenning and Skov, 2007; Jiménez-Alfaro et al., 2018). Therefore, in studies of large spatial scales, within which host species composition changes with geographical gradients, whether the observed biogeographic patterns of ECM fungal communities are related to the host biogeography or whether the ECM fungal community itself is responding to other factors, such as environmental and spatial factors, has not been always distinguished appropriately (Miyamoto et al., 2015; Matsuoka et al., 2016b). Therefore, in order to evaluate the effects of fungal response to environment or dispersal limitation on the geographic pattern of ECM fungal communities, studies focusing on single host species are indispensable.

When focusing on a single host species, especially those that are distributed across climatic zones, it is important to note that the genotypic structures of host species between geographically proximal sites can resemble each other. Distribution ranges of most tree species have expanded and moved from the refugia in the Last Glacial Maximum. Thus, genotypes of individual host tree species have their own geographic structure (e.g., genotypic composition and diversity), called phylogeography (e.g., Magri et al., 2006; Petit et al., 2012; Bemmels and Dick, 2018). Such phylogeographic patterns of host species (e.g., differences in genotypes across site) can also influence the ECM fungal communities, as some evidence show that ECM fungi co-migrate with their hosts (Kennedy et al., 2011; Séne et al., 2018) or they show preference to certain intra-specific genotypes (Gehring et al., 2017; Patterson et al., 2019). Thus, when the distribution patterns of ECM fungal communities correspond with host genetic pattern, there are two possible explanations for the correspondence: (1) Such patterns were generated in relation with their host species (e.g., co-migration) and (2) they were generated owing to the effects of direct fungal response to environment and dispersal limitation, independent to the host. However, so far, the relationships between geographic patterns of host genotypic structures and geographic distribution of ECM communities, and quantitative evaluation of the relative importance of the two possible explanations for the distribution pattern of ECM communities have been investigated in few studies.

We aimed to evaluate the geographic pattern of ECM fungal community and the effect of host genotypes and genotypic diversity on that pattern by focusing on a single host species *Castanopsis sieboldii* (Makino) Hatus. ex T.Yamaz. et Mashiba (Fagaceae). We chose *C. sieboldii* as the focal host species because this species is widely distributed across different climate regions of the Japanese archipelago and its phylogeographic pattern over its distribution range has been already documented with genetic markers (Aoki et al., 2014). Importantly, unlike Europe and North America, ice sheets were not present in the Japanese archipelago during the Quaternary period, and this, together with the complex mountainous terrain of Japan, resulted in the establishment of tree refugia in multiple regions across Japan (e.g., Tsukada, 1985; Ooi, 2016). Therefore, the present distributions of individual genotypes and genetic diversity of single species are assumed to reflect not only the environmental gradients like climate but also its migration history (Tsukada, 1985; Aoki et al., 2014). For these features, we considered *C. sieboldii* in Japan as the appropriate host species to evaluate the effect of host genotypes and genotypic diversity on the geographic distribution of the associated ECM fungal community. We specifically hypothesized that the richness and composition of ECM fungal communities change with geographic gradient (i.e., latitude and longitude) and that the variations in ECM communities are explained by geographic variations in host genotype variables and host genetic diversity variables. We further quantified the relative effects of environmental factors (i.e., soil property and climate) and spatial factors on the geographic pattern of ECM fungal community to identify the effects of host genetic variables. Also, we characterized the scale of spatial clustering (i.e., distance decay of similarity, Bahram et al., 2013) in the ECM fungal compositions across the study sites.

## Materials and Methods

### Study Sites and Sampling Procedure

**FIGURE 1:**
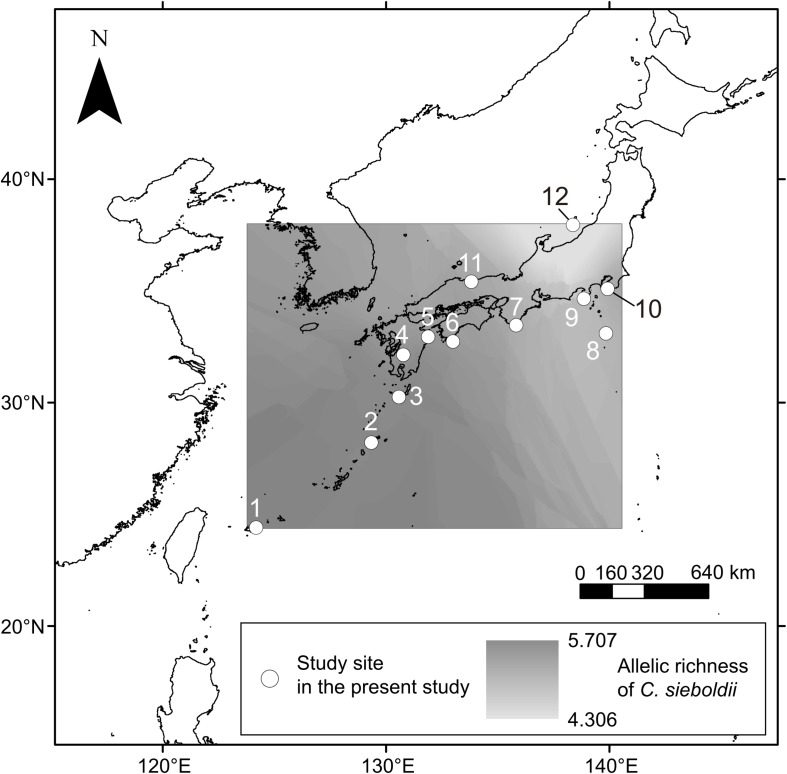
Sampling sites in this study. Numbers are consistent with site number listed in Table 1. Axes indicate latitude°N (y) and longitude°E (x). The estimated spatial pattern of allelic richness of *C. sieboldii* across sampling site was shown. See main text for details on the allelic richness.

We focused on an ECM host tree species *Castanopsis sieboldii* (Fagaceae), a dominant, canopy, and climax species in the Japanese *Castanopsis*-type evergreen broad-leaved forests. These *Castanopsis*-type forests characterize the biodiversity and endemism of the subtropical and warm-temperate zone in Japan. In Japan, 13 ECM tree species (Fagaceae) can appear in the same habitat with *C. sieboldii* including *C. cuspidata*, nine *Quercus* species (*Q. phillyraeoides*, *Q. gilva*, *Q. acuta*, *Q. hondae*, *Q. sessilifolia, Q. glauca*, *Q. salicina*, *Q. myrsinifolia*, and *Q. miyagii*), *Castanea crenata*, and two *Lithocarpus* species (*L. edulis* and *L. glaber*). Sampling was conducted in 12 mature *Castanopsis*-dominated forests (Table 1 and Figure 1) covering almost the entire distribution range of *C. sieboldii*, from Site 1 in Ryukyu Islands (∼25°N) adjacent to the present southern boundary of the host species’ distributional range to Site 12 in Sado Island (∼38°N) adjacent to its northern boundary. We selected the study sites based on following criteria although details in histories of the individual site are unknown: (i) Forest canopy is closed and forest contains at least 20 individuals of *C. sieboldii* with diameter at breast height (DBH) > 20 cm and (ii) there is no sign of large-scale natural and anthropogenic disturbance. Latitudes and longitudes of the sampling sites were strongly correlated (Pearson’s *r* = 0.845, *P* < 0.001), as the Japanese Archipelago is elongated from northeast to southwest. Sampling was conducted once for each site in the summer (from July to early September) in 2011, 2012, or 2013. At each site, sampling was conducted in <1 ha stands dominated only by *C. sieboldii*. We selected 20 individuals of *C. sieboldii* (DBH > 20 cm), which were not on slope, and collected a block of fermentation-humus (FH) layer (10 × 10 cm, 10 cm in depth) including tree roots within 3 m from each tree trunk. The soil blocks were collected within the expanse of the canopies of each individual *C. sieboldii* tree without canopies of other ECM trees and the sampling points were at least 3 m from each other. The blocks were stored in plastic bags and frozen at −20°C during transport to the laboratory. A total of 240 blocks (12 study sites × samples from 20 individual of *C. sieboldii*) were used for the study.

**TABLE 1:** The location and environmental factors of the study sites.

**Site No.**	**Site name**	***n*^1^**	**Latitude (°N)**	**Longitude (°E)**	**Sampling month**	**pH^2^**	**WC^2^**	**CN^2^**	**MAT (°C)**	**MAP (mm)**	**t2w (°C)**	**p2w (mm)**	**Distance to nearest Aoki’s site (km)**
1	Ishigaki	20	24.4178	124.1881	July. 2013	4.10 ± 0.36	29.7 ± 5.6	17.8 ± 2.0	24.4	2130.0	29.8	217.0	0.2
2	Amami	20	28.2225	129.3412	July 2013	3.24 ± 0.31	30.9 ± 14.6	21.8 ± 4.0	21.9	2420.5	28.6	0.0	8.5
3	Yakushima	20	30.2575	130.5817	July 2013	3.45 ± 0.41	65.1 ± 13.6	21.3 ± 2.5	20.4	3186.8	26.5	239.5	15.5
4	Hitoyoshi	20	32.1491	130.7803	July 2012	4.16 ± 0.14	48.6 ± 10.5	14.0 ± 1.7	15.8	2535.3	24.7	460.0	64.5
5	Saeki	20	32.9594	131.8913	July 2012	3.52 ± 0.57	56.8 ± 12.4	17.1 ± 1.8	16.8	2260.8	25.1	216.5	97.8
6	Ashizuri	16	32.7447	133.0002	July 2012	3.88 ± 0.28	59.5 ± 13.9	16.0 ± 2.2	18.4	2585.3	26.1	75.0	0.3
7	Kii	19	33.4653	135.8333	August 2011	3.57 ± 0.26	41.0 ± 11.5	15.6 ± 1.4	17.4	2700.0	27.0	35.0	23.0
8	Hachijo	20	33.1076	139.8414	August 2012	4.20 ± 0.45	49.7 ± 8.7	15.8 ± 1.0	18.0	3324.8	26.5	9.5	1.8
9	Izu	20	34.6509	138.8526	August 2011	3.61 ± 0.22	34.3 ± 8.5	16.7 ± 1.1	16.8	1748.6	25.6	51.5	5.3
10	Chiba	18	35.0934	139.9162	August 2011	4.02 ± 0.35	21.6 ± 7.9	14.4 ± 2.2	16.1	1918.0	26.7	9.0	20.0
11	Kurayoshi	20	35.4248	133.8224	September 2012	3.61 ± 0.22	35.2 ± 13.7	18.5 ± 2.4	14.9	1791.2	27.4	23.0	51.7
12	Sado	20	37.9661	138.3680	September 2013	3.78 ± 0.27	41.6 ± 8.4	18.2 ± 2.7	13.8	1846.1	25.5	146.0	9.9

*^1^Number of sample used for analyses (see text), ^2^Mean ± S.D. pH pH of FH (fermentation-humus) materials, WC water content of FH materials, CN C/N ratio of FH materials, MAT mean annual temperature, MAP mean annual precipitation, t2w mean daily temperature during the 2 weeks before each sampling date, p2w cumulative amount of precipitation during 2 weeks before each sampling date.*

In the laboratory, FH materials in the blocks were slightly melted at room temperature (20–25°C) and gently loosened. Then fine roots of trees were extracted from the samples using a 2-mm mesh sieve and gently washed with tap water to remove soil particles and debris. The sieved FH materials were used for the measurement of chemical properties as described below. In each block, 20 individual root segments (approximately 5 cm in length) were selected, and one ectomycorrhizal root tip (1–2 mm in length) was collected from each root segment under a binocular microscope at 20 × magnification. The 20 root tips were pooled for each block and kept in a microcentrifuge tube containing 70% ethanol (w/v) at −20°C. Before extracting DNA, the root tips were washed to remove small particles on the root surface by 0.005% aerosol OT (di-2-ethylhexyl sodium sulfosuccinate) solution (w/v) and rinsed with sterile distilled water. The root tips were then transferred to tubes containing cetyltrimethylammonium bromide (CTAB) lysis buffer and stored at −20°C until DNA extraction.

### DNA Extraction, PCR Amplification, and Pyrosequencing

The methods of DNA analysis were generally according to those described in Matsuoka et al. (2016a). Whole DNA was extracted from pooled root tips in 240 samples using the modified CTAB method described by Gardes and Bruns (1993). For direct 454 sequencing of the fungal internal transcribed spacer 1 (ITS1), we used a semi-nested PCR protocol. The ITS region has been proposed as the standard fungal barcode (Schoch et al., 2012). First, the entire ITS region and the 5′-end region of large sub unit (LSU) were amplified using the fungus-specific primers ITS1F (Gardes and Bruns, 1993) and LR3 (Vilgalys and Hester, 1990). Polymerase chain reaction was performed in a 20 μl volume with the buffer system of KOD FX NEO (TOYOBO, Osaka, Japan), which contained 1.6 μl of template DNA, 0.3 μl of KOD FX NEO, 9.0 μl of 2 × buffer, 4.0 μl of dNTP, 0.5 μl each of the two primers (10 μM) and 4.1 μl of distilled water. The PCR conditions were as follows: an initial step of 5 min at 94°C; followed by 23 cycles of 30 s at 95°C, 30 s at 58°C for annealing, and 90 s at 72°C; and a final extension of 10 min at 72°C. The PCR products were purified using ExoSAP-IT (GE Healthcare, Little Chalfont, Buckinghamshire, United Kingdom) and diluted by adding 225 μl of sterilized water. The second PCR was then conducted targeting the ITS1 region using the ITS1F fused with the 454 Adaptor and the eight base-pair DNA tag (Hamady et al., 2008) for post-sequencing sample identification and the reverse universal primer ITS2 (White et al., 1990) fused with the 454 Adaptor. The PCR was performed in a 20 μl volume with the buffer system of KOD Plus NEO (TOYOBO), which contained 1.0 μl of template DNA, 0.2 μl of KOD Plus NEO, 2.0 μl of 10 × buffer, 2.0 μl of dNTP, 0.8 μl each of the two primers (5 μM), and 13.2 μl of distilled water. The PCR conditions were as follows: an initial step of 5 min at 94°C; followed by 28 cycles of 30 s at 95°C, 30 s at 60°C, and 90 s at 72°C; and a final extension of 10 min at 72°C. The PCR products were purified with ExoSAP-IT and quantified with Nanodrop. Amplicons were pooled into five libraries and purified using an AMPure magnetic bead kit (Beckman Coulter, Brea, CA, United States). The pooled products were sequenced in five 1/16 GS-FLX sequencer (Roche 454 Titanium) at the Graduate School of Science, Kyoto University, Japan.

### Bioinformatics Analyses

The procedures used for bioinformatics analyses followed those described in Matsuoka et al. (2016a). In pyrosequencing, 315,685 reads were obtained. These reads were trimmed with sequence quality (Kunin et al., 2010) and sorted into individual samples using the sample-specific tags. The remaining 205,261 reads were deposited in the Sequence Read Archive of DNA Data Bank of Japan (accession: DRA004281). The pyrosequencing reads were assembled using Claident pipeline v0.1.2013.08.10 (Tanabe and Toju, 2013; software available online^1^), which is a highly parallelized extension of the Minimus assembly pipeline (Sommer et al., 2007). We removed the short reads (<150 bp) and then removed potentially chimeric sequences and pyrosequencing errors using UCHIME v4.2.40 (Edgar et al., 2011) and algorithm in CD-HIT-OTU (Li et al., 2012), respectively. After these filtering procedures, 130,004 reads were obtained. Reads from seven samples that had less than 50 filtered reads, were not used in the following analyses because such low read numbers could lead to the underestimation of ITS richness. Thus, the remaining 129,725 reads from 233 samples were used for further analyses. The number of sequencing reads per sample ranged from 50 to 3058 (mean, 551). All sequences were assembled across the samples using Assams at a threshold similarity of 97%, which is widely used for fungal ITS region (Osono, 2014), and the resulting consensus sequences represented molecular operational taxonomic units (OTUs). Then singleton OTUs were removed. Consensus sequences of the OTUs are listed in (Supplementary Table S1).

To systematically annotate the taxonomy of the OTUs, we used Claident v0.1.2013.08.10 (Tanabe and Toju, 2013), built upon automated BLAST-search by means of BLAST+ (Camacho et al., 2009) and the NCBI taxonomy-based sequence identification engine. Using the reference database from INSDC for taxonomic assignment, sequences homologous to ITS sequence of each query were fetched, and then taxonomic assignment was performed based on the lowest common ancestor algorithm (Huson et al., 2007). The results of Claident and the number of reads for the OTUs are given in Supplementary Table S1. To screen for ECM fungi, we referred to reviews by Tedersoo and Smith (2013) to assign OTUs to the genera and/or families that were predominantly ECM fungi. The resultant ECM fungal OTUs (ECM OTUs) were used for further analyses (see Supplementary Table S1).

### Environmental and Host Genetic Data

To investigate relationships between environmental factors (i.e., edaphic, climatic, and host biogeographic variables) and ECM fungal community (richness and composition of ECM OTUs), we measured and calculated the following variables (Table 1 and Supplementary Method S1). For each sample, water content (WC), pH, and carbon (C) and nitrogen (N) concentrations of the FH materials were measured. Carbon and nitrogen concentrations were determined by the combustion method using automatic gas chromatography (Sumigraph NC-22, Sumika Chemical Analysis Service, Ltd., Tokyo, Japan). The methods for WC, pH, and C and N concentrations followed those described in Matsuoka et al. (2016a). We calculated four climatic variables for each site: the mean annual temperature (MAT) (10-year averages before each sampling date), the mean annual precipitation (MAP) (10-year averages before each sampling date), the mean daily temperature during the 2 weeks before each sampling date (t_2__*w*_), and the cumulative amount of precipitation during the 2 weeks before each sampling date (p_2__*w*_). Data of air temperature and rainfall were obtained from the nearest station of the Automatic Metrological Data Acquisition System (Japan Meteorological Agency) to each study site. All stations are located within 8 km from each study site.

For evaluation of effects of the host genotypes and genotypic diversity on the ECM community, we used three host genotype variables (STRUCTURE clusters) and three host genetic diversity variables (allelic richness, frequencies of rare alleles, and frequency of private alleles) that were estimated from the data published by Aoki et al. (2014). Aoki et al. (2014) conducted phylogeographic analysis with 32 microsatellite primers (expressed sequence tags-simple sequence repeats, EST-SSRs), targeting 63 populations of *C. sieboldii* in Japan. Membership probabilities of three STRUCTURE clusters were used as host genotype variables. STRUCTURE analysis is a model-based Bayesian clustering approach to estimate genetic differentiation based on multi-locus genotype data (Pritchard et al., 2000; Falush et al., 2003). This method has been widely used for many population genetic and phylogeographic studies (e.g., Tsuda et al., 2015; Zinck and Rajora, 2016). Aoki et al. (2014) have reported that three genetic clusters were clearly detected within *C. sieboldii*, which are distributed in Ryukyu, western Japan, and eastern Japan, respectively (see also Supplementary Method S1). The combination of these host genotype variables can represent the genetic differentiation pattern within the host species at each sampling site. The three host genetic diversity variables were allelic richness, frequencies of rare alleles, and frequency of private alleles. Allelic richness is a measurement of mean number of alleles per locus after rarefaction method (El Mousadik and Petit, 1996). This indicator has been widely used for phylogeographic studies because it can evaluate genetic diversity correctly without being affected by differences of sample size (Widmer and Lexer, 2001; Zeng et al., 2015; Fischer et al., 2017). The percentage of rare (less than 1% in total) and private (unique to a single population) alleles represent genetic uniqueness. Rare alleles show relatively broad sensitivity and private alleles indicate high uniqueness. Because rare (including private) alleles are more sensitive to genetic drift than more frequent ones (Nei et al., 1975), they are easily removed from populations by bottleneck effects during range reduction in glacial periods. Historical stabilized populations such as refugia during glacial periods show high genetic uniqueness, and admixture populations derived from multiple refugia indicate high genetic diversity and low genetic uniqueness (Petit et al., 2003; Provan and Bennett, 2008). Therefore, considering these three host genetic diversity variables and three host genotype variables mentioned above, we could evaluate the effect of host genotypes and genotypic diversity. The data in Aoki et al. (2014) could not be directly used in the present study because our 12 study sites were different from those of Aoki et al. (2014). Therefore, we estimated host genetic variables in our study sites by the spatial interpolation in GIS software, which is commonly used with genetic variables (e.g., François et al., 2008; Ohtani et al., 2013; Worth et al., 2014; Wang et al., 2017). The minimum, maximum, and average distances from our sampling sites to Aoki’s nearest site are 0.2 km (Site 1), 97.8 km (Site 5), and 24.9 km, respectively (Table 1). Details of data processing and all host genetic variables are described in Supplementary Method S1 and Supplementary Table S2. The estimated spatial pattern of allelic richness of *C. sieboldii* was also shown in Figure 1. The correlations between latitude and longitude, and each environmental variable are provided in Supplementary Table S3.

### Data Analyses

The presence or absence of the ECM OTUs was used for all data analyses as binary data regardless of the number of 454 reads, because there are known issues with quantitative use of read numbers generated from amplicon sequencing (Amend et al., 2010; Elbrecht and Leese, 2015). All analyses were performed using R v. 3.0.1 (R Development Core Team, 2013).

Differences in the sequencing depth of individual samples affect the number of OTUs retrieved, often leading to the underestimation of OTU richness in those samples that had low sequence reads. In our dataset, because the rarefaction curves for 13% of samples did not reach asymptotes (Supplementary Figure S1), we conducted coverage-based rarefaction (Chao and Jost, 2012) to cover 96% of the total diversity of each sample. These rarefied numbers were used in the analyses of OTU richness. The rarefied OTU numbers were strongly related to the raw OTU numbers per sample (Pearson’s *r* = 0.890, *P* < 0.001, Supplementary Table S4).

To examine the biogeographic pattern of the rarefied ECM OTU richness, Pearson’s correlations between geographic variables, or latitude and longitude, and OTU richness were evaluated. Then we analyzed the relationship between the ECM OTU richness and the environmental variables using a generalized linear model (GLM). Error structure and link function of the GLM was Poisson and log, respectively. Environmental variables included 10 variables, that is three edaphic variables (pH, C/N ratio, and WC), four climatic variables (MAT, MAP, t2w, and p2w), and three host genetic diversity variables (allelic richness, frequency of rare alleles, and frequency of private alleles). The best model to explain the variation in OTU richness was chosen using the backward model selection based on Bayesian information criterion (BIC). For all variables in the selected model, partial pseudo-*R*^2^ were calculated according to Nagelkerke (1991).

To examine the biogeographic pattern of the ECM OTU composition, correlations between geographic variables, or latitude and longitude, and ECM OTUs composition were evaluated using the Mantel test with 9999 permutations. Geographic variables and presence/absence data of ECM OTUs for each sample were converted into a dissimilarity matrix using Euclidian distance and the Raup-Crick dissimilarity index (‘raupcrick’ command in the vegan R package), respectively. The Raup-Crick dissimilarity index is a probabilistic index and is less affected by the species richness gradient among sampling units in comparison to other major dissimilarity indices such as Jaccard’s and Sørensen’s indices (Chase et al., 2011). Then community dissimilarity of ECM OTUs among sites was ordinated using non-metric multidimensional scaling (NMDS). Presence/absence data of ECM fungal OTUs for each site were merged and converted into a dissimilarity matrix using the Raup-Crick index. Correlation of NMDS structure with geographic coordinates (latitude and longitude) and environmental factors (edaphic and climatic variables in Table 1 and host genotypic variables in Supplementary Table S2) were tested by permutation tests (‘envfit’ command in the vegan package, 9999 permutations).

We used variation partitioning based on the distance-based redundancy analysis (db-RDA, ‘capscale’ command in the vegan package) to quantify the contribution of the edaphic, climatic, host genetic, and spatial variables to the community structure of ECM fungal OTUs. The relative weight of each fraction (purely and shared fractions and unexplained fractions) was estimated following the methodology described by Peres-Neto et al. (2006). For the db-RDA, the presence/absence OTUs data for each sample were converted into a Raup-Crick dissimilarity matrix, and we constructed four models, including edaphic, climatic, host genetic, and spatial factors. The detailed methods for variation partitioning are described in Matsuoka et al. (2016a). First, we constructed edaphic, climatic, and host genetic models by applying the forward selection procedure (999 permutations with an alpha criterion = 0.05) of Blanchet et al. (2008). The full models were as follows: edaphic model [pH + C/N ratio + WC]; climatic model [MAT + MAP + t2w + p2w]; and host genetic model [three variables of cluster probability]. Then, we constructed the models using spatial variables extracted based on Moran’s Eigenvector Maps (MEM, Borcard et al., 2004). The MEM analysis produced a set of orthogonal variables derived from the geographical coordinates of the sampling locations. We used the MEM vectors that best accounted for autocorrelation and then conducted forward selection (999 permutations with an alpha criterion = 0.05, full model contained six MEM variables). Based on these four models, we performed variation partitioning by calculating adjusted *R*^2^ values for each fraction (Peres-Neto et al., 2006).

To characterize the scale of spatial clustering in the ECM OTUs compositions, Mantel correlogram analysis was performed (‘mantel.correlog’ command in the vegan package). A Mantel correlogram draws a graph in which Mantel correlation value *rM* is plotted as a function of the spatial distance classes. A positive (and significant) *rM* indicates that for the given distance class, the multivariate dissimilarity among samples is lower than expected by chance (Borcard and Legendre, 2012). Raup-Crick dissimilarity matrix of ECM OTUs community was used for the analysis. *P*-values were generated by permutational Mantel test (999 permutations) and adjusted using the Benjamini–Hochberg procedure (Benjamini and Hochberg, 1995) to correct the significance level for multiple comparisons.

## Results

### Taxonomic Assignment

In total, the 129,725 filtered pyrosequencing reads from 233 samples were grouped into 611 fungal OTUs with 97% sequence similarity (Supplementary Table S1). Among them, 365 OTUs (92,651 reads) belonged to ECM fungal taxa, with 351 OTUs being Basidiomycota and 14 OTUs being Ascomycota. Two hundred sixty-six and 43 OTUs of the 365 ECM OTUs were assigned with genus and species level, respectively (Supplementary Table S1). Each site yielded 36–64 OTUs (an average of 48 OTUs, Supplementary Table S4). At the family level, 365 ECM OTUs belonged to 19 families, and the common families were Russulaceae (134 OTUs, 36.7% of the total numbers of ECM fungal OTUs), Thelephoraceae (94 OTUs, 25.8%), and Cortinariaceae (45 OTUs, 12.3%). These three families accounted for 54.0–87.0% of total richness of ECM fungal OTUs at each site (Supplementary Figure S2). The most common OTU was *Cenococcum geophilum* (OTU_1), accounting for 44 of 233 samples (10 of the 12 sampling sites), followed by *Lactarius* sp. (OTU_563, 33 of 233 samples and 9 of the 12 sites), *Clavulina* sp. (OTU_603, 29 of 233 samples and 8 of the 12 sites), and Thelephoraceae sp. (OTU_602, 28 of 233 samples and 10 of the 12 sites). No ECM OTUs were detected from all the 12 sites. Whereas, 153 OTUs (41.9%) of the 365 ECM OTUs were detected in only one sample. Among the remaining 212 OTUs, which were detected in more than one samples, 172 OTUs were found in less than four sampling sites (Supplementary Figure S3) and these OTUs include those detected only in geographically proximal sites. For example, *Tomentella* sp. (OTU_81) and *Russula* sp. (OTU_591) were found only from the South-West islands (Site 1, 2, and 3, Supplementary Table S1) and *Tuber* spp. (OTU_248, OTU_ 134, and OTU_422) were found only in one sampling site.

### OTU Richness of ECM Fungi

The OTU richness significantly increased with latitude (Pearson’s *r* = 0.2078, *P* = 0.0019, *n* = 233) and longitude (Pearson’s *r* = 0.1991, *P* = 0.0030, *n* = 233). The model selection of the GLMs by BIC concluded that the model with host allelic richness + p2w + MAP + MAT was the best model accounting for the variation in OTU richness (Supplementary Table S5). The best model showed that the OTU richness increased with host allelic richness and with MAP and decreased with MAT and with p2w. Among the four environmental variables, MAT had the strongest relation with the OTU richness (Deviance = 40.29, pseudo-*R*^2^ = 0.1687, Table 2).

**TABLE 2:** The best generalized linear model (GLM) for the effects of environmental variables on rarefied ECM fungal OTU richness based on Bayesian information criterion (BIC).

	**df**	**Deviance**	**Coefficient**	**Partial pseudo-*R*^2^**
Host allelic richness	1	2.65	0.5554	0.0121
p2w	1	9.94	−0.0015	0.0446
MAP	1	7.27	0.0003	0.0328
MAT	1	40.29	−0.1264	0.1687
Residual	229	237.53		

*MAT mean annual temperature, MAP mean annual precipitation, p2w cumulative amount of precipitation during 2 weeks before each sampling date.*

### Community Structures of ECM OTUs

**FIGURE 2:**
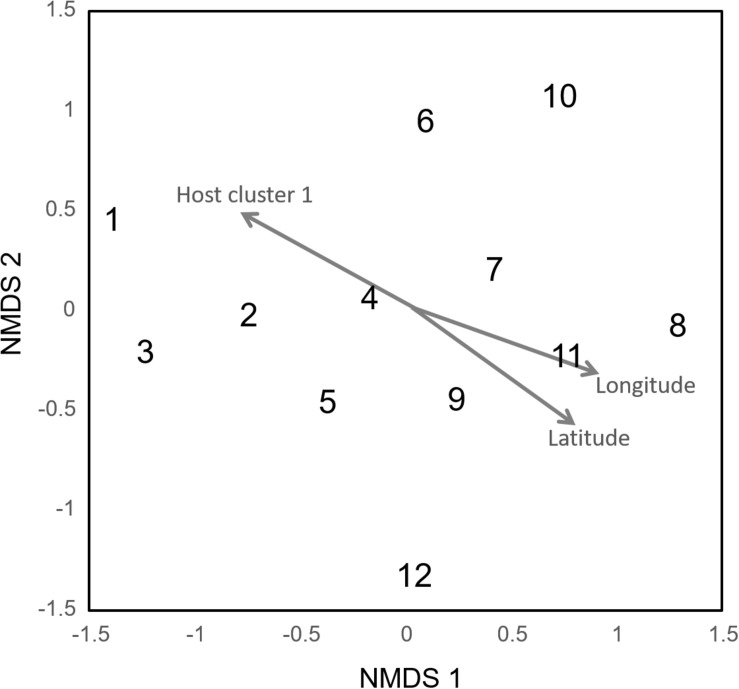
Community dissimilarity among the sites as revealed by non-metric multidimensional scaling (NMDS) ordination (stress value = 0.173). Numbers are consistent with site numbers listed in Table 1.

The Mantel test and NMDS ordination revealed the biogeographic changes in the ECM fungal compositions. The dissimilarity of ECM fungal community among samples was significantly and positively correlated with distances calculated from latitudes (Mantel’s *r* = 0.1054, *P* = 0.0001) and longitudes of sites (Mantel’s *r* = 0.1397, *P* = 0.0001). The NMDS plot showed the geospatial change of ECM OTU composition among sites (Figure 2, stress value = 0.173). The ordination was significantly correlated with latitude and longitude (‘envfit’ function; latitude, *R*^2^ = 0.636, *P* = 0.010; longitude, *R*^2^ = 0.715, *P* = 0.006) and a host genotypic variable (cluster 1, *R*^2^ = 0.694, *P* = 0.008) but not significantly with other edaphic, climatic, and host variables (*P* > 0.05).

**FIGURE 3:**
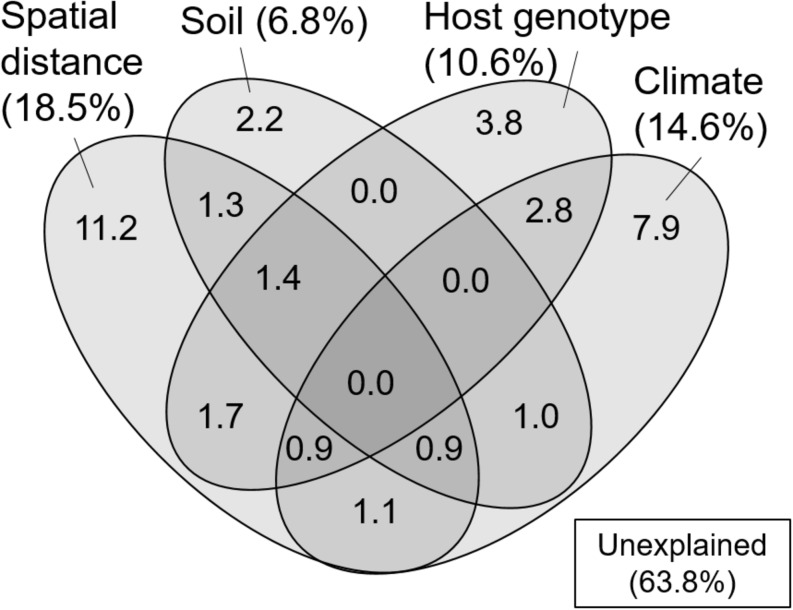
Venn diagram showing pure and shared effects of spatial distance, soil, host genotype, and climate on the ECM fungal community composition as derived from variation partitioning analysis. Numbers indicate the proportions of explained variation.

In the variation partitioning, pH, C/N ratio, and WC of the FH materials were selected as edaphic factors, MAT, MAP, t2w, and p2w were selected as climatic factors, three STRUCTURE variables were selected as host genetic factors, and four MEM vectors (vector 4, 5, 1, and 2) were selected as spatial factors. The percentages explained by the edaphic, climatic, host genetic, and spatial fractions were 6.8, 14.6, 10.6, and 18.5%, respectively. Pure fractions of soil, climate, host, and space were 2.2, 7.9, 3.8, and 11.2%, respectively (Figure 3). Among the shared fractions, spatially structured host genetic fraction (i.e., shared fraction between space and host, 4.0%) and climatically structured host genetic fraction (i.e., shared fraction between climate and host, 3.7%) were the two largest fractions (Figure 3). In total, 36.2% of the community variation was explained and the remaining 63.8% were unexplained.

**FIGURE 4:**
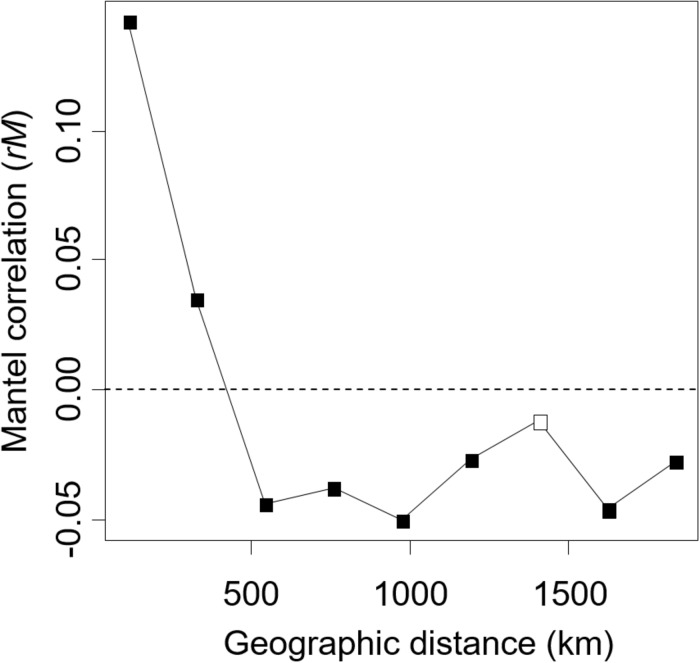
The Mantel correlogram shows the extent of spatial autocorrelation of the ectomycorrhizal fungal community composition. Filled and open boxes indicate the significant and non-significant correlation at 5% level, respectively.

In the Mantel correlogram, in which data were lumped into nine distance classes for this analysis, the first two distance classes exhibited significant spatial autocorrelation of the ECM fungal community (*P* < 0.05, Figure 4). The first and second distance classes represent 108 and 325 km, respectively. No significant positive autocorrelations were found for the longer distance classes (*P* > 0.05).

## Discussion

In the present study, we successfully demonstrated the biogeographic pattern of the ECM fungal community associated with single host species (*C. sieboldii*) and detected the correlations between host genetic variables and ECM communities. Our results suggest that the biogeographic pattern of ECM fungi could be partly affected by the host phylogeography, which reflects the historical migration and distribution expansion of the host species. The populations of *C. sieboldii* were divided into three genetic clusters, one in the South-West Islands, another in Western, and the other in Eastern Japan (Supplementary Method S1 (Figures M1-1–M1-3); Aoki et al., 2014). These clusters indicate that, during the Last Glacial Maximum, major refugia existed in these three regions separately and *C. sieboldii* expanded its distribution from these refugia (Aoki et al., 2014).

The correlation between the genetic clusters of *C. sieboldii* and OTU composition of ECM fungi could be partly due to the past migration of ECM fungi following the expansion of their host range (i.e., co-migration) after the last glacial period. In this case, the OTU composition of ECM fungi could show spatial structure reflecting the spatial structure of genotype composition of *C. sieboldii* (i.e., geographically closer populations share similar genotype composition). Such “host mediated” spatial structure may be included in the shared fraction between host and spatial variables in variation partitioning (Figure 3). Such co-migration of ECM fungi with their host has been reported in some previous studies, for example, current patterns of genetic relatedness in the Périgord truffle (*Tuber melanosporum*) seem to mirror patterns of post-glacial revegetation in the French Alps (Murat et al., 2004). Although mechanisms on co-migration or co-dispersion are not completely clarified, dispersals of tree seeds carrying ECM spores have been reported (Séne et al., 2018). Alternatively, the preference of ECM OTUs to particular host genotypes (Gehring et al., 2017; Patterson et al., 2019) could also explain the relationship between host genotype and ECM composition. That is, the difference in genotype corresponds to the difference in some traits such as drought tolerance, and ECM fungi respond to these trait differences (Gehring et al., 2017; Patterson et al., 2019). The fraction explained only by host genetic variables in variation partitioning may include such genotype preference of ECM fungi, which is irrespective of spatial distance. Nevertheless, the *C. sieboldii* trait preference of the associated ECM fungi was evaluated only in a few studies, and the importance of preference of ECM species to particular host genotypes on ECM communities has not been fully clarified (Bubner et al., 2013; Lang et al., 2013).

A positive correlation was found between OTU richness and the host’s genetic diversity (i.e., allelic richness). In a group with high allelic richness, it can be inferred that a large population size has been maintained for a long time or different populations have merged (Petit et al., 2003). For *C. sieboldii*, the genetic diversity was higher in sites around the three regions where refugia were speculated to be present (Supplementary Method S1, and see above). In these sites, ECM fungal communities may have been stably maintained for a long period, providing opportunities for repeated speciation, and thus, may have yielded high species diversity. It can also be possible that the ECM OTU richness increased due to the mixing of ECM fungal communities that originated from other host populations derived from different refugia. That is, when ECM fungal communities are following the range expansion of *C. sieboldii*, at sites where different populations meet (and thus different fungal communities meet), ECM fungal diversity would become high as fungi from different communities merge. However, fungal diversity was low in low latitude region where one of the refugia was supposedly present (Supplementary Table S4). Nevertheless, the results of GLM indicated that climatic factors should have stronger effect on ECM fungal OTU richness than host genotypic diversity.

### ECM Fungal Compositions and the Related Factors

In the present study, we successfully demonstrated that environmental filtering and dispersal limitation on fungi themselves (i.e., not via their host) can generate the biogeographic structure of the ECM community, by quantifying the effects of environmental and spatial factors on the ECM community relative to the effects of host genetic variables. Thus far, the effect of host phylogeography on biogeographic patterns of ECM communities has rarely been quantified, suggesting that patterns of ECM communities explained solely by environmental and spatial factors may also be affected by host phylogeography. Indeed, in variation partitioning, some parts of environmental and spatial fractions were shared with host genetic variables (Figure 3). This indicates that environmental and spatial factors may partly affect the ECM community via host response to environment and dispersal, and previously demonstrated effects of these factors might be overestimated. Nevertheless, not only such shared fractions but also environmental (soil and climatic) and spatial fractions, which are not shared with the host genetic fraction, explained the difference in ECM community composition. This implies that environmental filtering and spatial processes such as dispersal limitation (Peay et al., 2012; Peay and Bruns, 2014) on fungi themselves can generate the biogeographic pattern of ECM fungal community independent of the host phylogeography. Especially, the fractions explained by spatial variables alone and by climatic variable alone explained a larger proportion of the ECM composition compared to edaphic and host genetic fractions (Figure 3). This result suggests that the biogeographic pattern of ECM community composition associated with *C. sieboldii* could be primarily structured by spatial processes reflecting historical dispersal events and by climatic filtering. Our observations are consistent with recent findings that biogeographic patterns of ECM fungal community (including ECM fungal mycelium and/or propagules in soils) are explained by spatial distance (Talbot et al., 2014; Glassman et al., 2015) and/or climatic variables (Bahram et al., 2012; Miyamoto et al., 2015; van der Linde et al., 2018). We also detected a distance decay pattern of the ECM composition in the range up to c.a. 300 km, indicating that the ECM composition changes with increasing spatial distance (Figure 4). The spatial pattern is caused by both the dispersal limitation of ECM fungi and environmental variables that change with spatial distance (Bahram et al., 2013). Results of variation partitioning suggest that both dispersal (pure spatial fraction in Figure 3) and environments (shared fractions between space and environments in Figure 3) may influence the ECM community. In our study sites, dispersal distances seem to be limited not only in the hypogeous taxa [e.g., *Tuber* spp. (OTU_248, OTU_ 134, and OTU_422 in Supplementary Table S1)] that are prone to isolation by distance, but also in the epigeous fruiting groups, which may affect the geographic structure of ECM community. However, knowledge of the scale of fungal dispersal is limited. In the future, therefore, it is necessary to investigate the dispersal distance of fungi and the factors that cause the spatial structure, as well as the specific spatial scale of distance decay pattern.

It is important to note that the causal relationship between host genotypes and ECM communities cannot be concluded from our observation, and other (measured and unmeasured) environmental variables, which vary with the host variables can also lead the correlation between host and ECM. Similarly, the spatial fraction may also result from the effects of unmeasured environmental variables that are spatially structured (Borcard and Legendre, 1994). In addition, approximately 64% of the variation remained unexplained (Figure 3). Several factors may be invoked to explain this unexplained variation. For example, environmental factors that were not measured in the present study (e.g., soil potassium content, Tedersoo et al., 2014), biotic interaction with other fungal and/or surrounding tree species (Hubert and Gehring, 2008; Bogar and Kennedy, 2013; McBurney et al., 2017), and stochastic processes driven by ecological drift and dispersal including island biogeography (MacArthur and Wilson, 1967; Hubbell, 2001) may be plausible explanations of the unexplained variation. For example, there are ECM tree species that inhabit only in the South-West islands [e.g., *Quercus miyagii* (Fagaceae) in Site 1 and 2]. In Japan, there are 13 ECM tree species, which can appear in the same habitat with *C. sieboldii* (see the section Materials and Methods). These tree species may influence the geographic pattern of ECM fungal community, although our sampling was carried out in forest stands dominated by *C. sieboldii* alone. In addition, since the *Castanopsis*-type forests have a long cultural history, such as protections of vegetation by shrines, human activities may have influenced the genotype flow of *C. sieboldii*, although forests apparently influenced by such human activities were not selected as our sampling sites and any phylogeographic patterns of *C. sieboldii* affected by human activities were not detected (Aoki et al., 2014). The present study may also underestimate the explanatory power of spatial factors because our sampling sites were too sparse to detect potential spatial structures of ECM fungi in our *Castanopsis* forests. The reasons are that (i) approximately half of the OTUs in more than two samples were detected only in one sampling site, and (ii) in the result of the mantel correlogram, the distance decay of the ECM composition rapidly decreased from the first distance class (up to approximately 100 km) to the second distance class (up to approximately 300 km), which is short considering the distances between sampling sites (Figure 1). These unmeasured factors and relative importance of environmental variables and spatial distance should be tested in future studies.

### OTU Richness of ECM Fungi

We observed that OTU richness decreased at lower latitudes. From the results of GLM, this geographic pattern of OTU richness could be attributed partly to climatic factors, especially temperature and precipitation (Table 2 and Supplementary Table S3). Operational taxonomic unit richness decreased at sites with high mean annual temperature (MAT). This pattern is consistent with the result from a meta-analysis of global patterns of ECM communities (Tedersoo et al., 2012), which showed that ECM fungal richness decreases at tropical and subtropical regions. This lower richness in tropical and subtropical region may be related to the difference in thickness of the soil organic layer. In low latitude regions, soil organic layer is thinner than that in high latitudinal regions because of active organic matter decomposition, which can make vertical habitat segregation of ECM fungi difficult (Dickie et al., 2002; Tedersoo et al., 2012). Indeed, it was reported from a previous study that, in Japan, the soil organic layer is thinner in sub-tropical forests than temperate forests (Osono, 2015), though in *C. sieboldii* forests, such comparison has never been conducted.

The GLM results showed that OTU richness could be related to precipitation. First, the OTU richness decreased with cumulative amount of precipitation during the 2 weeks before each sampling date (p2w). The potential effect of water stress (e.g., the availability of oxygen in soil decreases with increasing precipitation) on the OTU richness is discussed as a reason for the negative influence of rainfall (e.g., Tedersoo et al., 2012). In our study sites, p2w had a wide range from 0 to 400 mm (Table 1), and water stress could affect OTU richness in the sites with high p2w. Whereas, MAP showed a positive relationship with OTU richness, though the explanatory value was lower than p2w. A negative relationship between OTU richness and MAP at a global-scale was reported by Tedersoo et al. (2012), which is contradictory to our result. The reason for this inconsistency is unclear, but the difference in the range of MAP may partly have contributed to this difference. The MAP value in our study (1,700–3,300 mm) was higher than that of the previous study (mostly around 0–2,000 mm), and this might have led to a different response of the community owing to their adaptation to high precipitation.

## Conclusion

In the present study, we demonstrated that the ECM fungal community associated with *C. sieboldii* showed a biogeographic structure that was correlated with the host genotypic structures, by focusing on a single host species and considering its intraspecific genetic richness and genotypes. This result suggests that the phylogeography of host species (i.e., the history of migration and environmental responses) can affect the associated ECM fungal communities and their biogeographic patterns. Moreover, with quantitative analysis, we showed that dispersal limitation and climate responses of fungi themselves, not via their host responses, could strongly affect the biogeographic pattern of the ECM fungal community. These results emphasize the importance of considering host phylogeography as well as fungal environmental responses and spatial processes (dispersal and colonization limitation) when studying the biogeographic patterns of ECM fungal community. Further studies are required to confirm whether similar patterns can be observed in other host species or climatic regions and to clarify how the relationship between intraspecific variation of host and ECM fungal community is generated.

## Data Availability Statement

All data of the 454 sequencing were shared in DRA (Accession Number: DRA004281).

## Author Contributions

SM designed the study. SM and TO designed and carried out the fieldwork. SM, YS, and EK performed the experiments. SM, TI, and YS analyzed the data and interpreted the results. SM, YS, HD, TI, and TO wrote the initial draft of the manuscript. EK critically reviewed the manuscript.

## Conflict of Interest

The authors declare that the research was conducted in the absence of any commercial or financial relationships that could be construed as a potential conflict of interest.

## Footnotes

1 https://www.claident.org/
